# ‘It’s not just about me’: a qualitative study of couples’ narratives about home death when one of the partners is dying of cancer

**DOI:** 10.1177/26323524231189517

**Published:** 2023-08-02

**Authors:** Margareta Aurén-Møkleby, Lisbeth Thoresen, Anne Marit Mengshoel, Kari N. Solbrække, Gunvor Aasbø

**Affiliations:** Institute of Health and Society, University of Oslo, Postboks 1089 Blindern, 0317 Oslo, Norway; Institute of Health and Society, University of Oslo, Oslo, Norway; Institute of Health and Society, University of Oslo, Oslo, Norway; Institute of Health and Society, University of Oslo, Oslo, Norway; Institute of Health and Society, University of Oslo, Oslo, NorwayDepartment of Research, Cancer Registry of Norway, Oslo, Norway

**Keywords:** cancer, caregivers, couples, end-of-life care, home, home death, narrative analysis, palliative care, patient and partner dyads, preference for place of death

## Abstract

**Background:**

Most cancer patients state a preference for home death. Care and support from primary caregivers are important to enable dying at home. A preference for home death from the perspective of couples has rarely been investigated.

**Objectives:**

To explore how a preference for home death is understood and enacted in couples where one of the partners is dying of cancer.

**Design:**

A qualitative interview research design with a narrative approach was used.

**Methods:**

Five couples participated in dyad interviews. During the analysis, two interviews that particularly illuminated couples’ shared and individual views were chosen as the primary cases.

**Results:**

The interviews show, in two highly different ways, how a preference for home death is a significant relational matter. The interviews are presented as two cases: ‘Struggles in an Unknown Terrain’ and ‘Reliance at the Kitchen Table’. They show how a preference for home death can be understood and enacted as a struggle or as reliance based on the couple’s shared biography and the partner’s ability to care for the partner during the end-of-life phase. The analysis highlighted the negotiations that underpin a preference for home death. In these negotiations, the couples drew on idealised understandings of home death. These ideas were supported by cultural values related to autonomy and independence as well as participation and citizenship. Thus, in the negotiations about being cared for and caring, legitimate dependency and the maintenance of a reciprocal relationship were balanced. The presence of healthcare professionals and medical devices in the home had to be balanced with the need to maintain a sense of self and an authentic home.

**Conclusion:**

A relational perspective on a preference for home death made us attentive to couples’ negotiations. These negotiations give couples the opportunity to re-evaluate and reconfirm individual and mutual needs in the end-of-life phase.

## Introduction

‘Being at home is lovely. Dying at home must be wonderful’. These words belong to a woman dying of cancer and were spoken during an interview for this study in which she participated with her husband. The woman wanted to spend her last days in her home, which she treasured, surrounded by her loved ones. For her, the home was the preferred place of death. This preference is consistent with what most cancer patients want, as reported in various countries.^1–3^ To die at home resonates with ideas of more human, social and ‘natural’ ways of dying, as well as cultural and political norms and ideas of autonomy and self-governance.^4^ In Norway, where this study was carried out, a recent white paper^5^ stated that terminally ill people wanting to die at home should have this option. However, there is a gap between this preference and the actual place of death among cancer patients.^3^ In Norway, most cancer patients die in healthcare institutions;^6^ in 2021, for example, 83% died in institutions and 16% died in private homes.^7^ Although dying and death are mainly managed by healthcare services, the ‘Report of the Lancet Commission on the Value of Death’^8^ points to how most day-to-day care is handled by the dying person and their primary caregiver(s). Cancer patients living with a life partner are more likely to have a preference for home death;^2^ they are also more likely to die in a preferred place compared with individuals living on their own.^9^ This pattern highlights the importance of having a dedicated primary caregiver, preferably a spouse or a partner, when one is seriously ill and wants to be at home in the last phase of life. Life partners are entwined with each other’s lives; they are connected through shared experiences and acts of reciprocity. These relationships create meaning in life^10^ and, as this study will show, also in death. In this article, we explore the preference for home death from a relational perspective. Specifically, we investigate how such preference is understood and enacted among couples who share a home.

### Background

Dying and death are extraordinary and existential matters that remain ‘unknown’^11^ despite always being anchored to culture, history and place. Because death is such an exceptional, value-loaded event, the physical areas where dying happens become *places* – that is, areas with considerable meaning and significance. The meaning of places, such as private homes, is often related to personal identity, family, events and relationships.^12^ Historically, death used to be a collective responsibility and an integrated part of life, which mostly happened at home.^13^ Starting in the middle of the 20th century, as the role of family, community and institutions in the West changed, caring for the dying was moved from private homes to institutions.^8^ Scholars have highlighted how, due to this change, dying has gradually become more private and hidden.^14^ Death itself has become a solitary and medicalised experience^15^ governed and directed by healthcare professionals.^13^ The professionalisation of dying and death has resulted in a loss of laypersons’ knowledge and ability to deal with death, in contrast to what was the case for earlier generations.^8,16,17^

Cancer is a leading disease-related cause of death worldwide^18^ and the most common disease-related cause of death in Norway.^7^ Many people live with metastatic illnesses that have complex symptoms and treatments and involve complicated care decisions and uncertainty.^19^ Typically, the terminal stage of cancer disease is characterised by increased weakness, decreased appetite, fatigue, immobility, confusion and drowsiness.^19^ Even though the characteristics of the terminal stage are well described, predictions about the disease trajectory in individual patients are difficult even for experienced palliative care physicians.^20^ Accordingly, dying from cancer may therefore, as we understand it, entail a rather unpredictable trajectory for patients and their partners.

In Norway, public specialised and primary health care services provide palliative and end-of-life care.^5^ In recent decades, the municipality’s responsibility for palliative care has increased.^21^ Palliative care should primarily take place in and close to the patient’s home.^22^ For patients living at home, the ambition is that municipal home care services (MHCS) are provided and tailored to the needs of patients and their informal caregivers, ideally in close collaboration with the general practitioner and palliative care teams in the specialist health care services.^5,21,22^ But it has been reported that palliative care is insufficiently integrated in health care services in many Norwegian municipalities.^21^ However, in the case of cancer, several municipalities provide additional services to patients and their families to coordinate services and provide information and guidance throughout the illness trajectory.^22^

The presence and care offered by informal caregivers are crucial for seriously ill people to spend time at home and die at home. According to Norwegian health care policy, patients should receive more specialist health services in their own home,^23^ making the role of informal caregivers even more essential. The informal end-of-life care is often related to practical and medical care as well as social and emotional support.^24,25^ There is a substantial body of research on care and support provided by primary caregivers to seriously ill family members.^10,26–29^ The same is true of patients’ and primary caregivers’ experiences of the end-of-life phase.^25,30–32^ However, studies specifically focusing on couples in the end-of-life context and on their preference for home death are rare. So far, we have not found any. We argue that individual autonomy regarding a preference for home death needs to be complemented by a relational perspective. In our case, this means paying attention to how reciprocity, individual and mutual care, and needs are negotiated between the partners when one of them is dying from cancer and wishes to die at home.

In this article, we use a narrative approach to analyse couples’ stories and unpack the complexity of home death. The aim of the study is to expand our understanding of preferences for home death from a relational perspective by answering the following research question: How is a preference for home death understood and enacted in couples’ narratives when one of the partners is dying of cancer?

## Methods

### Study design

This is a qualitative interview study conducted with couples in which one of the partners is seriously ill with cancer and prefers home death. The study adopts a narrative approach^33,34^ focusing on what is said and how couples talk about and handle the preference for home death both together and as individuals. Conducting narrative interviews allows scholars to collect experience-centred stories and detailed accounts.^33^ The couples were interviewed together. Dyadic interviewing offers couples the chance to construct a common narrative out of their individual experiences of a shared phenomenon^35,36^ – in this case, the preference for home death. Being interviewed together, the participants will be influenced by each other’s talk and positions,^33^ and there is a risk of unbalanced participation where one participant can be more prominent than the other.^35,36^

### Recruitment and participants

We recruited adult couples aged 18 years and older. The couples had been informed about the short life expectancy of the patient, who had stated a preference for home death. Nurses from primary healthcare services in four different municipalities, as well as medical doctors in a specialist palliative care ward, helped to identify couples to recruit. The healthcare professionals introduced the study, and if a couple was interested, their contact information was given to the first author. From September 2021 to October 2022, five couples were included and interviewed. The cancer patients – three women and two men – were aged between 59 and 79 years. They had been diagnosed with their current cancer between 1 and 13 years prior to the study. Two of them had metastatic disease. Two had previously been curatively treated for another cancer. The need for care in the home varied from once a week, daily, to several times a day. More complex care needs, such as administration of enteral feeding or care of stomas, required more assistance from MHCS. The prerequisite for a possible home death seemed to relate to symptom burden and the caring resources and capacities of the partner.

The partners – two women and three men – were aged between 62 and 76 years. All the couples had been married for at least 20 years.

Four of the patients died within 5 and 24 days of the interviews. At the time of writing this article, the fifth patient is still alive. The names of the participants used in this article are pseudonyms.

### The interviews

The couples were interviewed in their homes by the first author. As the patients had bothersome symptoms, such as short breath and tiredness, their partner assisted them during the interview. The interviewer focused on the couple as well as on each participant to facilitate an inclusive setting. The interviews lasted between 30 and 90 minutes and were audio recorded. An interview guide was used as an aide-mémoire. This included themes such as what they knew about home death, how home death had become a preference, how they managed everyday life, and their thoughts about the time ahead. The interviewer wrote short field notes after each interview, which included descriptions of and reflections on the interaction, how the patient was affected by the illness, and how the home was organised. The field notes supplemented the analysis of the interviews. The first author transcribed the interviews verbatim.

### Narrative analysis

A thorough initial reading of the transcripts showed how the couples talked about and dealt with home death in very different ways. To understand the interviews’ specificities and capture both the individual and shared perspectives, we carried out a case-oriented narrative analysis. This method is useful to uncover the forms of identity making and meaning making at stake in the research topic.^33^ In line with this choice, the stories presented here are based on interviews with two of the couples, Ava and Bernard and Denise and Charles. The generation of data was guided by the concept of information power, which relates to dialogue quality, sample specificity, and analysis strategy.^37^ By focusing our attention on *how* they told their stories and *what* they told,^33^ narrative analysis helped us to sharpen our understanding of how the preference for home death was a specific social and relational situation, as well as how the couples narrated the relationship between past and present life experiences.^38^ Narratives are often constructed through a plot which constitutes the nerve in a story.^38^ In the interviews, such construction was not always easy to recognise, as the couples seemed to live in an ‘in-between time’ merging past, present and future life. As will be demonstrated in the findings section, the two stories illuminate how the wish to spend time and die at home is talked about and handled by couples in complex and paradoxical ways.

The analytical focus of the article was developed jointly by the five authors and advanced by the first and last authors. The following analytical questions guided the analysis: What did the couples talk about in relation to the preference for home death, individually and as a dyad? How were the end-of-life phase and death talked about, and how did the participants inscribe themselves into that situation? Following Riesman,^38^ we paid attention to the use of words and concepts, how the participants interacted with each other and the interviewer, as well as the cultural, relational and personal resources available to the couples when narrating the preference for home death.

The research team discussed the analytical process as the study progressed, which allowed the team members to consider, challenge, and refine different understandings and interpretations.

### Ethical considerations

The first author – a PhD student and an experienced intensive care nurse – conducted the interviews. The participants knew of her professional background prior to the interview; hence, the interviewer’s clinical background might have reassured them that the interviews would be conducted in a considerate way. The couples chose to participate despite bothersome symptoms, demanding care responsibilities and limited time. The interview was sensitive for all those involved as talking about dying and death is challenging, even though the participants knew the topic to be discussed. Thus, the principle of not causing harm^39^ guided the planning, conduct, and analysis of the interviews. In the interview situations, the interviewer constantly balanced being attentive to the couples’ interaction and her positionality as a responsible researcher and caring nurse. After each interview, the interviewer debriefed the participants and encouraged them to contact their municipal home care nurse or cancer coordinator if questions or if existential or emotional reactions arose. The couples could also contact the interviewer to discuss matters related to the interview.

The study was approved by the Norwegian Data Inspectorate for Research (project no. 432421) and the Regional Committee for Medical and Health Research Ethics (project no. 95689). Before the interviews started, the couples signed an individual informed consent form. The audio recordings and transcripts were stored at the University of Oslo on a secure research platform that complies with the current privacy regulations. Only the authors had access to the material.

## Findings

In the two cases presented here, the telling rarely took place in a temporal order. On the contrary, the conversation went back and forth, which is a common way of talking when people tell stories.^40^ For this reason, to make the stories intelligible and present the findings coherently, some quotes consist of sequences from different parts of the interview; these instances are marked with an ellipsis within square brackets (i.e. [. . .]). Furthermore, to make the quotes easier to read, when a participant did not complete a sentence, we wrote implied words within square brackets. Within a quote, italicised text within square brackets describes certain actions, such as pointing and nodding.

### Struggles in an unknown terrain

Ava and Bernard are in their late sixties; they have been married for about 20 years and live in a three-room apartment. They both have an academic background and had careers in public authorities before retiring. Ava was diagnosed with cancer about a decade ago and lived well for many years. The prognosis is now poor and curative treatment has recently been stopped. She receives palliative care at home. Professionals from the MHCS visit several times per day; they administer enteral feeding and adjust medications when needed.

During the interview, Ava lies on the living room couch, struggling to find a comfortable position; she communicates constant feelings of sickness and fatigue. Bernard sits in an armchair; he is ready to assist Ava whenever she needs a change of position or something to drink. The medical devices – a wheelchair, a hospital bed, and medical pumps – are conspicuous and occupy considerable space. Ava now receives enteral nutrition via a percutaneous endoscopic gastrostomy (PEG) tube (a feeding tube placed through the abdominal wall and into the stomach to provide nutrition) as she can no longer eat regular food. Bernard also has some health issues, which limit how much he can take care of his wife. In their narrative, Ava’s determination and agency are significantly emphasised; they are seemingly a cornerstone in their past as well as their present everyday life. Ava presents herself as being used to speaking her mind and acting independently. Up until recently, she has been the one caring for the apartment, her family and friends, and Bernard. However, as her current situation and future home death are challenging to handle, Ava’s role as an independent individual is now partly compromised.

Once informed that she had little time left to live, Ava declared her preference for home death. She links this preference to a contemporary cultural narrative that helps her to set the scene. When speaking, Ava addresses both the interviewer and Bernard:

Ava: I don’t know anyone [who has died at home], but I’ve watched documentaries on TV where this is talked about. I think it’s a good possibility for those who can manage it, and I told the hospital that I wanted to die at home.

Interviewer: So, you brought up the issue of home death?

Ava: Yes, it’s usually me who brings things up. Right, Bernard?

Bernard: Yes, that’s right.

Ava: But I also said that it’s not just about me. It might be difficult for Bernard to cope, so I don’t know if it’s possible. But when I returned back home [from the hospital], I knew where I wanted to die. [I want to die] in there – in our bedroom. Not over there [*she points to the room with the hospital bed*], but in there [*she points to their ordinary bedroom*]. [. . .] For me, it was mostly that I could have my children and Bernard around me – on the bed or next to it. A little chat – if I’m able to – and then you can go out. But there are physical things that [are challenging], so I get it. It’s selfish. [. . .] Ideally, at the end, I want as few people [care professionals] here as possible, but there has to be someone here who sits discreetly in a corner. Someone who sees where this is going, and who gives you advice on how to manage the end. One [person] who takes up the least space but contributes the most.

In Ava’s narrative about realising a good home death, the image of an authentic home with limited signs of illness and unfamiliar carers is important. In line with this, the professional care that supports Ava and guides Bernard and the family has to be balanced – present but not visible. In Ava’s narrative, dying happens in a controlled way. However, her bothersome body and dependency on others, conveyed by Ava as ‘physical things that are challenging’, stand out as a hindrance to enacting such a home death. On one hand, her condition challenges the possibility of familiar interactions; this is reflected, for instance, in her difficulties in accepting the hospital bed as a deathbed. On the other hand, her husband struggles to handle the care requirements as she is now very ill and in need of almost constant care.

By narrating uncertainty and doubt each from their own perspective, Ava and Bernard constantly negotiate the realisation of home death:

Ava: I’m lying here waiting to die, but at the same time, I want to have a good life while I die. It’s exhausting. You use a lot of energy to stay well enough so you can stay at home. And when are you going to give up? Just lie down and give up? It’s a twofold issue. Being at home is lovely. Dying at home must be wonderful. I don’t know what to do. [I’m not sure] if he can do it.

Bernard: I have no problem accepting it [Ava dying at home], but I don’t know if I can manage the care. After all, a completely different physical follow-up is required in the end. I don’t have any experience of that. This will be completely new, like entering a completely unknown terrain. That’s why I feel insecurity and doubt. [. . .] For me, this is 24/7. The care professionals visit but things can happen between the visits. At the same time, it’s terribly tiring to have people – strangers – around you all the time, so there’s always that balance of being able to be a bit. . . You need to be by yourself. With people here. . . It becomes a bit intrusive. That’s why there is an uncertainty around this.

Ava: I see how this is tiring for Bernard. We are different types. I’m tired too, but you get tired in different ways from having people in and out of the house. And Bernard gets tired of it; I can see that. I care about him. I want it [home death], but am I selfish enough to push that through? I don’t know. Don’t think so. I think we have to accept. . . He can’t end up getting ill. We can’t have that. What do you think, Bernard?

Bernard: I think I can manage, but if there’s a lot of stress, I get a little tired, of course.

Ava says that spending time at home is physically strenuous as well as existentially challenging. She communicates uncertainty about the dying process and how Bernard will manage to care for her. Faced with an unpredictable illness trajectory, her earlier resoluteness and ability to act are of little help. She pictures giving up as a turning point in the dying process, possibly involving a change in the care she receives when she can no longer stay at home due to escalating symptoms and Bernard’s doubts and limited capacity. Ava describes dying at home as ‘twofold’, thus indicating an ambivalence towards the idea of the ‘good home death’ that happens in an authentic and peaceful way. This idea clashes with her increased need for care and dependency on others. Her ill and uncontrollable body causes uncertainties related to dying at home that question the narrative of the good home death.

Bernard narrates his insecurity by using the metaphor ‘unknown terrain’. He knows neither how to manage the ‘physical follow-up required’ nor what awaits them in Ava’s dying process. Is the terrain all downhill from now, or will the trajectory involve further ups and downs? Will there be any points of reference to manage Ava’s forthcoming death? The only concrete guidance is the one from the MHCS. Although Bernard knows that both Ava and he are dependent on their help and support, he experiences their presence as a burden that disrupts his feeling of home, sense of self and independence.

Throughout the interview, Ava negotiates the realisation of dying at home. Her wish and determination are mainly weighed against paying attention to and caring for her husband. Bernard, who describes his care responsibilities as ‘24/7’, may understate how demanding the care of Ava is. Between the visits of the MHCS, Bernard is on his own. He is ready to help his wife the best way he can, which leaves him with little respite. As he is also not in good health and lacks the required care experience, the terrain is not only unknown but also probably worrisome and overwhelming for him.

Initially, Ava and Bernard’s narrative displays an ‘I’ and a ‘you’ – that is, their story holds no clear resolution. However, towards the end of the interview, their shared story opens up, and a narrative plot may be interpreted; their shared story increasingly acknowledges their different needs in terms of care and support. During the narrative, the preference for home death is modified, and assuring mutual care is reflected as the critical point in the last phase of life. The two partners need a manageable situation where both are cared for and supported.

### Reliance at the kitchen table

Charles and Denise, in their early seventies and late sixties, respectively, have been married for the past 20 years and live in a house together. Before retiring, Charles had done manual work, including roles with considerable responsibility, while Denise had worked as a carer in healthcare institutions. Charles was first diagnosed with cancer some years ago; for a time, the illness had been kept at bay. However, at the time of the interview, he had metastases and a poor prognosis. Given his short life expectancy, palliative care in the home had been initiated. Charles received weekly visits from MHCS checking on medication. If needed, they were prepared to provide more care.

Charles sits with a cigarette and a coffee at the kitchen table, the place where they want to conduct the interview. As Charles is short-breathed, Denise does most of the talking. As demonstrated in their narrative, Charles takes part in the interview by nodding or through short comments, confirming what his wife says. However, if he disagrees with something, he expresses this. Therefore, both husband and wife participate and are involved, though Denise, with Charles’s approval, speaks on behalf of both. Throughout the interview, their ability to cope and handle illness and crisis is linked to the fact that, some years ago, Denise recovered from a serious disease. Although Charles does not talk much, he underlines how her recovery has been decisive for them as a couple and how the fact that ‘life has settled’ is of great importance, especially now that he is soon to die and Denise will carry on without him.

Despite knowing that he was critically ill, the information about his short life expectancy, which he received at the hospital some weeks ago, has made him deeply sad. It has taken some time for him to come to terms with the situation. Although his situation has worsened, Charles wants to stay at home, and Denise agrees with him. Serious illness and dying at home are not new for Denise; she has experienced them both within her family and as a professional carer. Based on this, she has a clear idea of the advantages of home as a place for dying; these can be summarised in the possibility of holding on to autonomy, governance and privacy. As she speaks, Denise addresses her husband and the interviewer:

Denise: [Sometimes] you struggle to breathe. Then we use morphine. I can see how his condition changes from day to day now. He had a seizure at home; since then, I think it has gradually worsened. It’s getting harder for you to walk as you don’t have the strength, and yesterday you also did not feel like eating, so you actually only ate breakfast. Yesterday. Because you could not bear dinner or anything. You said you had no appetite. Right?

Charles: That’s right. . .

Denise: [Now it’s about] having it the best possible way at home. None of us likes to be in a hospital, and I had the experience of my great-grandparents and my grandmother, who died at home, so I guess that’s part of it. At that time, there were no home healthcare services – people had to cope on their own. I think that influences both of us. End life at home, if possible. In peace and quiet with your loved ones present. The children can be here. All of us together. Those who want to. The children and maybe the grandchildren. [. . .] We’ve talked about it [home death] and decided together. We’re honest and mostly we agree; sometimes we argue a little, but not for long. We soon become friends again, but now we don’t talk that much. He mostly sleeps. We have the mornings here at the kitchen table, with coffee. You get a cigarette and something to eat, and then you want to lay on the couch to rest. [*Charles nods.*]

The prominent narrative voiced by Denise represents their shared story. Denise frequently addresses Charles directly to invite him into the conversation or for confirmation of what she says. Although their chances to talk and discuss are now limited, their story incorporates the couple’s strong sense of mutual reliance; they stand in this together.

The preference for, and agreement on, home death is something Charles and Denise share. So far, the situation is manageable. The significance of managing on their own is also highlighted by the references to memories of home death in the family. Avoiding hospitalisation seems important, which may reflect prevailing cultural understandings of institutions and hospitals as impersonal and alien places. In these environments, there is a risk of losing autonomy and control. Staying at home offers the couple the possibility of maintaining a sense of self and intimacy; for instance, through the routine of the cigarette and coffee at the kitchen table.

Their idea of the good home death is pictured as something peaceful and quiet, which includes members of the family. At home, they can gather the family and still have enough space for privacy. The narrative shows no doubt or insecurity regarding Charles dying at home. Nevertheless, a fundamental premise in Denise’s narration of Charles’s possible home death is her experience as a former healthcare professional. She has the knowledge of what to look for (e.g. his breathing and appetite), how to interpret what she sees (e.g. a seizure and reduced body function), and how to handle it (e.g. administrating drugs to ease pain and discomfort). Thanks to her competencies, the need for MHCS has been limited. However, when the interview was carried out, it was clear, at least to Denise, that she would soon need support in caring for Charles dying at home:

Denise: We may need more home medical supplies eventually.

Charles: Mmm. . . [*nodding*]

Denise: I know you don’t like to talk about this because it is not relevant today, but that time will come. The most important thing for us is that we take one day at a time and that we know there is help available when we need it. We must not be afraid to ask. Then again, he is from a generation that thought you should manage on your own and not bother others, but we do that now. We have learnt that we must do this together to make it happen – together with the team. Then, I think everything will turn out fine. Don’t you think?

Charles: Yes.

In their narrative and enactment of home death, the role and involvement of external help are under negotiation. Denise negotiates with Charles concerning not only their need for more support but also their idea, or Charles’s idea, of how home dying will be managed without help from others. Using ‘we’ in her negotiation, Denise emphasises their shared need for support. Possibly, she does this to make it acceptable to Charles and downplay his dependency on her and others. At the same time, she emphasises her need for support and relief. Letting others in involves a reassessment of the deeply rooted value and identity of being independent. Denise’s proactive attitude in seeking external help may reflect her professional background. She sees the importance of being prepared for more advanced care needs. In Charles’s view, this way of thinking may be both unnecessary and threatening. He relies heavily on Denise, and the future includes his death. By focusing on life, one day at a time, death is kept at a distance. His way of holding on to life, is not to talk about the progression of illness or plan for what lies ahead. Despite different understandings and acceptance levels related to the need for external care, the overall narrative of Charles and Denise presents a clearly defined ‘we’ that forms the basis of their preference for Charles’s home death.

## Discussion

This study’s findings show how the preference for home death is understood and enacted through ongoing negotiations in couples where one of the partners is dying of cancer. Negotiations concerning ideals, the relationship of care and the role of external help are central in the narratives of the couples. These three aspects surface more or less openly. Furthermore, embedded in these negotiations are cultural values and principles of autonomy and independence as well as reciprocity and mutual care. The negotiations are given meaning through the partners’ narratives, which encompass their past, present and future shared life. Negotiating appears important to re-evaluate and reconfirm individual and shared needs in the end-of-life phase and sort unsettled matters that may cause ambivalence close to the moment of death.

### Negotiating ideals

The narratives display how a preference for home death is not simply a decision about place; it is a desire that reflects ideals of the maintenance of self, autonomy and independence. This aspect shows that decisions related to location at the end of life are contextual, personal, relational, conditional and flexible processes, as pointed out by Gerber *et al.*^30^ Illness and dying disrupt familiar ways of thinking, being and acting in relation to the self and others.^41,42^ People facing death, both dying persons and their partners, are situated within dynamic processes that self-create their personal identity.^43^ But as our study demonstrates, understandings and enactments of a preference for home death might be intertwined with the need to preserve the continuity of the self and reciprocity – a sense of ‘we-ness’.^44,45^ As the cases show, notions of home death are often related to a wish to preserve independence and self-determination, virtues highly valued in Western societies. However, when people become highly dependent on care and support from others, these virtues are difficult to uphold.

The value of self-determination is reflected in what has been called ‘cancer citizenship’ and points to the insights of Broom *et al.*^46^ into how the present calls for awareness. The present, the authors argue, ‘ask[s] for complete conviction and commitment to live, to its fullest, whatever life is left’. Thus, in this context a good cancer citizenship involves planning, organisation and collaboration with several significant others.^46^ Good cancer citizenship, then, can be understood as an ideal that should be upheld alongside those of maintenance of the self, autonomy and independence. In our study, this ideal was expressed as the desire to have a good life while dying [despite the exhaustion] and plan for the time ahead [despite the worries]. However, grounding a preference for home death on nostalgic^47^ and romanticised^48,49^ ideals – aiming for ‘a good death’ – might lead to a sense of failure for both dying persons and their primary caregivers when it is impossible to achieve this.^8^ Based on these insights and how the preference for home death was negotiated in this study, we argue that reconstructing such preference – making it valid and applicable to people’s situations and life trajectories – can be a way to support dyadic governance and agreement in the end-of-life phase.

### Negotiating the needs of the cared for and the carer

Our analysis demonstrates the individual and shared understandings of the preference for home death. In particular, the couples narrated from an ‘I’ and a ‘you’ position or from a ‘we’ stance. This is similar to what Gardner^50^ found when exploring meaning in the end-of-life phase within a dyadic context among older couples, who went back and forth between individual and dyadic voices. The analysis in the current study displays that individual understandings, interests and needs are balanced and agreed upon through negotiation. This aspect underlines how narratives are constructed within specific contexts and become co-constructions of tellers and listeners when making meaning out of lived experience.^38^ However, in the end-of-life phase, negotiations take place in a complex and fragile context, which is overshadowed by the fact that one is soon to die. Chattoo and Ahmad^51^ address this kind of situation when they argue that ‘personal care involves negotiation of boundaries between notions of relatedness and legitimate dependence on one hand, and independence and integrity of the embodied self on the other’.^51^ This type of relational complexity resonates with our findings. In this study, the ambivalence related to the needs of both the cared for and the carer was balanced with autonomy and reciprocity as well as an idea of good cancer citizenship.

Our findings show that both care recipients and caregivers try to navigate their former roles with anticipated roles in the present and their limited future, individually and as a dyad. We understand the metaphor of the ‘unknown terrain’ expressed in the narrative of struggles to go beyond the mere practical management of a deteriorating illness. This metaphor may also signal a change of familiar roles in the relationship as well as a transformation of the home.

### Negotiating the presence of external help in the home

In the context of end-of-life care, the home can be experienced as a place where autonomy can be exercised^30,31,49^ or as a transformed place with decreased personal space, especially for the caregiver.^52^ This came to light also in our study. A preference for home death often draws on the idea of the authentic and familiar home. Therefore, for some, an altered home with a hospital-like environment may be alienating and too demanding. In many countries, including Norway,^5,53,54^ home death is a political matter. Those in favour of it state that people who want to die at home should have the option to do so. However, as noted by Neto,^48^ few people fully comprehend what dying at home requires, and family caregivers can often experience poor support in this matter.^48^ As today’s patients are sicker and often need more complex interventions and medical care, caring for a dying person at home is more complicated than in the past.^47^ Hence, help from MHCS is crucial not only to assist with care tasks but also to decide when home death is not possible.

In the narratives we have investigated, the role of MHCS was central. However, their presence in the home, as well as that of medical devices, although necessary, was experienced as disturbing and intrusive. This was linked to how this presence disturbed the authentic home or decreased personal space. The need for external help appeared to be an unsettled matter in the couples and caused ambivalence even close to death. Health care services may play an important role in making home death manageable through highly competent health care professionals and sufficiently tailored and timely provided palliative care.^5,19^ However, extensive palliative care provision in the home is not always sufficient to make home death manageable. As the narratives in this study display, home death can be perceived as manageable or unmanageable at an individual and relational level depending on the extent of the symptoms and the care abilities of the partner.

## Strengths and limitations

Recruiting couples for this study was challenging since the ill person had little time left and the topic of the interview may have been difficult to discuss for both the patient and their life partner. The dyad interviews took place shortly before the ill partner died. This setting made it possible to gain genuine insights into the couple’s dynamics regarding the preference for home death. Most of the literature on this topic is based on retrospective studies of bereaved partners or interviews with ill persons before the end-of-life phase. Therefore, we believe that our study shows important nuances and complexities in this field. The narratives were chosen to display the diversity of how couples understand and enact a preference for home death. Though the narratives are unique, they do reflect relational dynamics that are recognisable and applicable beyond the individual stories and their specific contexts. Therefore, the relational dynamics described in the couples’ shared narratives are transferable to other informal caring relationships in end-of-life care.

The following limitations of this study must be considered. The interviewed couples were all made up of white, elderly, and middle-class citizens of Norway. Hence, a more diverse sample in terms of age, sexual orientation (e.g. LGBTQ persons), cultural background and class (e.g. couples with a lived experience of poverty and deprivation) might have brought other narratives to the fore. The research team also consisted of white, middle-class female academics, and this might have represented a possible limitation in the study. The data were in Norwegian and the quotes were translated into English; thus, precise wording and nuances may have been changed or lost.

## Conclusion

In this study, a relational perspective on a preference for home death revealed the ongoing, multifaceted negotiations that enable couples to re-evaluate and reconfirm individual and mutual needs in the end-of-life phase. A relational focus also showed that, when a couple is affected by illness and the need to manage care responsibilities, the active support and recognition of the patient and the partner might differ significantly. Furthermore, a crucial condition for developing a relational focus in healthcare services is understanding the negotiations couples have regarding a preference for home death and the ideals this preference contains – maintenance of self, autonomy and independence. Home death involves the dying person and the partner, and health care professionals must support both not only to realise their wish but also to decide when home death is not possible.

## Declarations

**Ethics approval and consent to participate:** The Norwegian Data Inspectorate for Research (no. 432421) and the Regional Committee for Medical and Health Research Ethics (no. 95689) approved the study. All participants gave written consent to participate in the study.

**Consent for publication:** All participants gave written consent to publish from in this study.

**Availability of data and materials:** The audio recordings and transcriptions in Norwegian of the dyad interviews from the study ‘The Role of Close Relations in Cancer Patients’ Decisions on Home Death: An Exploratory Study’ (University of Oslo, Norway) are stored on a platform that complies with current privacy regulations. The data that support the findings of this study are available in Norwegian upon request from the corresponding author. The data are not publicly available due to privacy or ethical restrictions.
